# Strain to evaluate ventricular function in Fontan patients undergoing exercise cardiac magnetic resonance imaging

**DOI:** 10.1186/1532-429X-16-S1-P123

**Published:** 2014-01-16

**Authors:** Shafkat Anwar, Ravi Doddasomayajula, Marc S Keller, Matthew A Harris, Ajit Yoganathan, Mark A Fogel, Kevin K Whitehead

**Affiliations:** 1 Pediatrics, Cardiology, The Children's Hospital of Philadelphia, Philadelphia, Pennsylvania, USA; 2 Biomedical Engineering, Georgia Institute of Technology, Atlanta, Georgia, USA; 3 Pediatrics, Radiology, The Children's Hospital of Philadelphia, Philadelphia, Pennsylvania, USA

## Background

Feature tracking strain (FTS) is a new technique to assess cardiac function from cardiac magnetic resonance (CMR) images. We compared FTS with conventional function parameters in single ventricle subjects with Fontan physiology undergoing exercise CMR.

## Methods

28 Fontan subjects (median age 16.6 years, 14/28 morphologic left ventricle, 14/28 morphologic right ventricle) underwent a resting and exercise CMR. Standard high resolution, segmented balanced steady state free precession (SSFP) images were acquired with breath holds at rest. Cartesian real time (RT) cine images were acquired at rest then repeated at exercise, targeting the HR at anaerobic threshold on a recent metabolic exercise stress test (EST). Ventricular volumes and cardiac output were calculated offline. Off-line strain analysis was performed (TomTec 2D CPA, v. 1.0) on the "4-chamber" and short-axis views at basal, mid, and apical levels.

## Results

**Figure 1:**
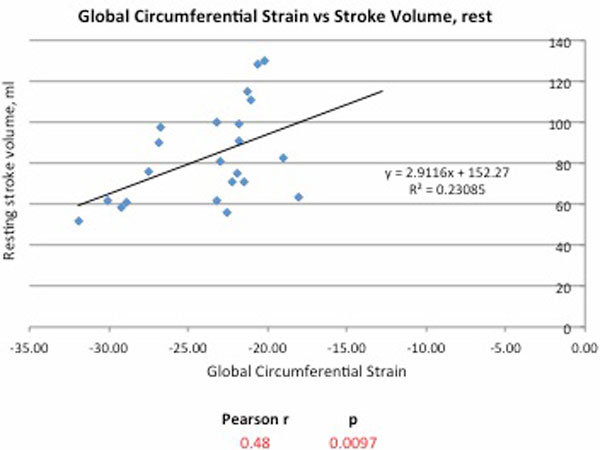


**Figure 2:**
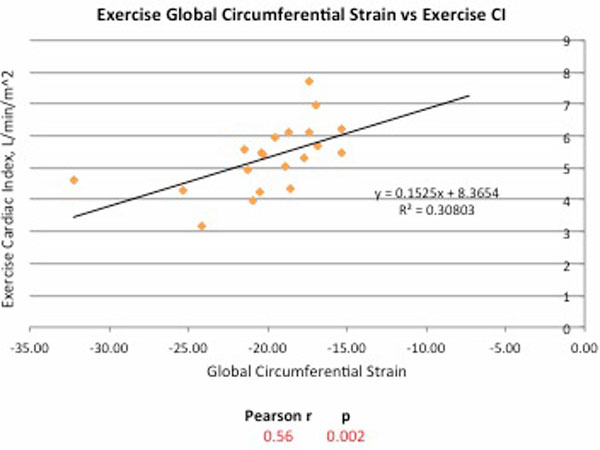


At rest there was moderate correlation between global circumferential strain (GCS) and stroke volume (SV), Pearson r = 0.48 (p = 0.009) and cardiac index (CI), r = 0.39 (p = 0.04), Figure 1. There was also good correlation between resting GCS and exercise SV, r = 0.56 (p = 0.002). During exercise there was good correlation between GCS, SV and cardiac output indexed to body surface area (SV/BSA, CI), r = 0.45 (p = 0.015) and r = 0.56, p = 0.002) respectively, Figure 2. There was no relationship between GCS and ejection fraction, end-diastolic volume or end-systolic volume at rest or exercise.

## Conclusions

In single ventricle Fontan patients there is moderate correlation between strain and measures of ventricular output (SV and CI) at rest and exercise, but not directly to ventricular size or ejection. Further studies are required to determine the role of feature-tracking strain in the assessment of ventricular function. Analysis of regional strain may helpful in understanding myocardial mechanics in the single ventricle.

## Funding

Dr Kevin K. Whitehead: NIH K23 Grant HL089647 from the National Heart, Lung and Blood Institute. Dr. Mark Fogel: NIH R01HL098252-01, "Understanding mechanisms of Fontan failure and key predictors for patient outcome."

